# The Speed of Thought

**Published:** 1883-06

**Authors:** 


### THE SPEED OF THOUGHT.

We have several times given the readers of the *Journal* a report of what has been done by scientists to determine the rate at which nervous influence is transmitted through the telegraphic system of our bodies. Some recent investigations on the subject are thus summed up in the *American Journal of Arts and Sciences.*

Sensations are transmitted to the brain at a rapidity of 180 feet, or at one-fifth the rate of sound; and this is nearly the same in all individuals.

The brain requires one-tenth of a second to transmit its orders to the nerves which preside over voluntary motion; but this amount varies much in different individuals, and in the same individual at different times, according to the disposition or condition at the time, and is more regular the more sustained the attention.

The time required to transmit an order to the muscles by the motor nerves is nearly the same as that required by the nerves of sensation to pass a sensation; moreover it passes nearly one-hundredth of a second before the muscles are put in motion.

The whole operation requires one and one-fourth to two-tenths of a second. Consequently when we speak of an ardent, active mind, or of one that is slow, cold or apathetic, it is not a mere figure of rhetoric, but an absolute and certain fact that such a distinction, with varying graduation, really exists.

The method by which these nerve motions are measured is thus described:

If a cylinder divided into 360° be caused to rotate 1,000 times in a second, it is evident that the passage of one of those degrees before a given point is equal to the 1-360,000th part of a second; this may be divided by a microscope, so that a period of time equaling the ten-millionth, or even the one hundred-millionth, part of a second may be measured. By this arrangement it is possible to measure the rate of nervous impulse. Suppose an electric shock be given to the arm; it produces a sensation and a contraction of the muscles; then by noting the interval of time between the shock and the contraction of the muscles, the time occupied by the action of the brain to produce the contraction, however quick, will be ascertained. By trying this experiment on various parts of the body, the amount of sensibility of the different leading muscles may be determined.—*Boston Journal of Chemistry.*

